# Life-Threatening Hyponatremia Secondary to Chronic Kratom Use: A Case Presentation

**DOI:** 10.7759/cureus.29073

**Published:** 2022-09-12

**Authors:** Gabriella Martin, Dylon P Collins, Harol Valenzuela

**Affiliations:** 1 College of Medicine, Nova Southeastern University Dr. Kiran C. Patel College of Osteopathic Medicine, Fort Lauderdale, USA; 2 Nephrology, Florida Kidney Physicians, Port Charlotte, USA

**Keywords:** kratom, pain modulation, osmotic demyelination, drug-induced hyponatremia, severe hyponatremia

## Abstract

Hyponatremia is defined as a serum sodium concentration of less than 135 mEq/L. Severe hyponatremia is defined as a serum sodium concentration of less than 125 mEq/L and is a life-threatening complication that must be managed promptly to avoid irreversible neurological damage. One particular cause of hyponatremia is the ingestion of recreational drugs, such as 3,4-Methylenedioxymethamphetamine (MDMA), also known as Ecstasy. Another drug with limited understanding of its adverse effects on specific individuals and is widely available to purchase legally is Kratom (*Mitragyna speciosa*). Here, we present the case of severe hyponatremia secondary to the ingestion of Kratom. Kratom is believed to act on various pain-modulating receptors and may explain its role in causing hyponatremia. Unfortunately, Kratom remains poorly understood and underreported. Our case illustrates the need for further in-depth studies to determine the complete toxic profile of Kratom, providing awareness to clinicians in anticipation of severe complications that may develop.

## Introduction

Serum sodium concentration is tightly regulated on a physiological level between 135-145 mEq/L. Hyponatremia is defined as a serum sodium concentration less than 135 mEq/L and is dependent on the ratio of total body water (TBW) to total body solutes (TBS), as first proposed by Edelman in 1958 [1,2]. 

The extracellular space determines volume status and can explain how an imbalance of TBW to TBS leads to hyponatremia. Causes of hypovolemic hyponatremia include gastrointestinal fluid loss, diuretic use and mineralocorticoid deficiency [3]. Causes of euvolemic hyponatremia include the syndrome of inappropriate antidiuretic hormone (SIADH), Addison's disease, and hypothyroidism [3]. Causes of hypervolemic hyponatremia causes include chronic renal failure, congestive heart failure, and cirrhosis [3]. Once the volume status has been determined, further steps to consider in the workup of hyponatremia are calculating the serum osmolality, urine osmolality, and urine sodium concentration. The main symptoms of hyponatremia are neurologic, which stem from electrolyte imbalances causing cerebral edema. Acute hyponatremia occurs in less than 48 hours and tends to present with more severe neurologic presentations, whereas patients with chronic hyponatremia tend to have an adaptive mechanism of generating idiogenic osmoles to maintain an asymptomatic state [4]. 

Many drugs can cause hyponatremia, both legal and illegal. Of particular interest are substances that have been used for many years yet remain poorly studied and lack understanding of acute or chronic adverse effects. Kratom has over 25 distinct metabolically active alkaloids with side effects that remain underreported and are yet to be extensively studied for their efficacy and safety [5]. Kratom can be obtained legally and acts on receptors similar to those of opioids. A few reported adverse effects that have been demonstrated are hypothyroidism, hypogonadism, hepatitis, acute respiratory distress syndrome, posterior reversible encephalopathy syndrome, seizure, and coma [5]. Understanding Kratom's role as the primary or secondary cause of these adverse effects is clinically significant to the future management and regulation of the drug. Here, we present the case of a 62-year-old male who was found to be ingesting large doses of Kratom and subsequently developed life-threatening hyponatremia of 103 mEq/L and was aggressively managed.

## Case presentation

A 62-year-old male presented to the emergency department at a local hospital with a three-day history of altered mental status (AMS) and restlessness. During that time, he reportedly had little food or fluid intake. The patient's past medical history consists of only chronic alcoholism. On admission, his vitals were stable, and there were no signs of trauma. On examination, the patient was obtunded and did not respond to sternal rubs or verbal commands. His eyes were equal and reactive to light, and he had equal breath sounds bilateral. A clinical exam determined his volume status to be euvolemic. A complete blood count (CBC) and comprehensive metabolic panel (CMP) were ordered. The hemoglobin measured 13.4 g/dL (13.5-17.5 g/dL), white blood count 7.73 g/dL (4.5-11 g/dL), hematocrit 33.9% (34-52%), platelets 254,000 mm3 (150,00-450,00 mm3), sodium 103 mEq/L (135-145 mEq/L), potassium 3.5 mEq/L (3.5-5.0 mEq/L), bicarbonate 25 mEq/L (22-28 mEq/L), chloride 62 mEq/L (95-107 mEq/L), creatinine 1.84 mg/dL (0.6-1.2 mg/dL), blood urea nitrogen (BUN) 21 mg/dL (8-21 mg/dL), glucose 157 mg/dL (70-100mg/dL), magnesium 1.5 mEq/L (1.5-2.0 mEq/L), albumin 4.1 g/dL (3.5-5.5 g/dL) and calcium 9.5 mg/dL (8.4-10.2 mg/dL). Further tests showed urine sodium of less than 110 mEq/L, urine osmolality of 243 mOsm/kg (50-1400 mOsm/kg), normal thyroid function, and serum cortisol levels above 60. A head computed tomography (CT) scan showed no significant cranial changes. The patient was immediately started on 3% sodium chloride at 30 mL/hr, supplemented with magnesium sulfate, folic acid, and thiamine. He was transferred to the intensive care unit (ICU) for continued management. The serum sodium target goal during the first 24 hours was set between 107-109 mEq/L to avoid overcorrection by more than 6 mEq/L. Serum sodium levels were checked every two hours.

The following day the patient was conscious and aware of his name, surroundings, and medical situation. He had overcorrected by 4 mEq with a morning serum sodium of 113 mEq/L. The 3% sodium chloride was discontinued, and 2 mcg of DDAVP (desmopressin) twice daily injections along with a 400 mL bolus of D5W (5% dextrose in water) were administered. A new target serum sodium was set at 114-116 mEq/L for the next 24 hours.

On day three, the patient's serum sodium was 116 mEq/L, and a new target sodium of 122-124 mEq/L was set for the following 24 hours. The DDAVP injections were stopped, and the patient was only administered D5W as needed for overcorrection. Later that day, a bag of Kratom was found in possession of the patient, and after a detailed discussion, it was determined that the patient had a history of taking large daily doses of the substance. The patient was consulted on avoiding the substance, as it might have led to his hyponatremia.

**Figure 1:**
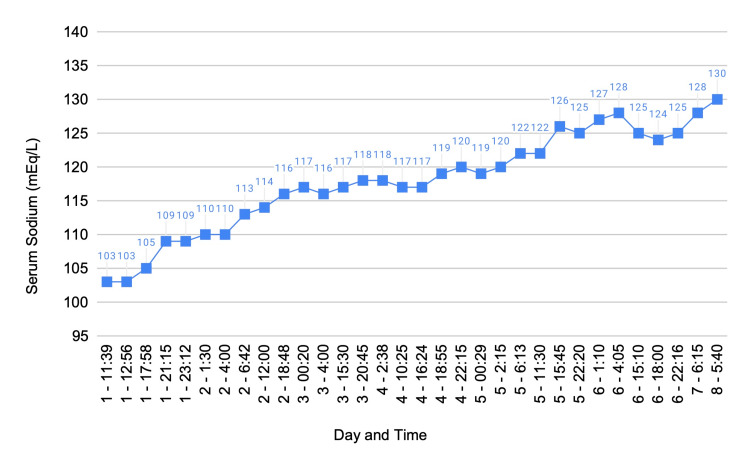
Serum sodium (mEq/L) from day one of admission to day eight of discharge

**Figure 2:**
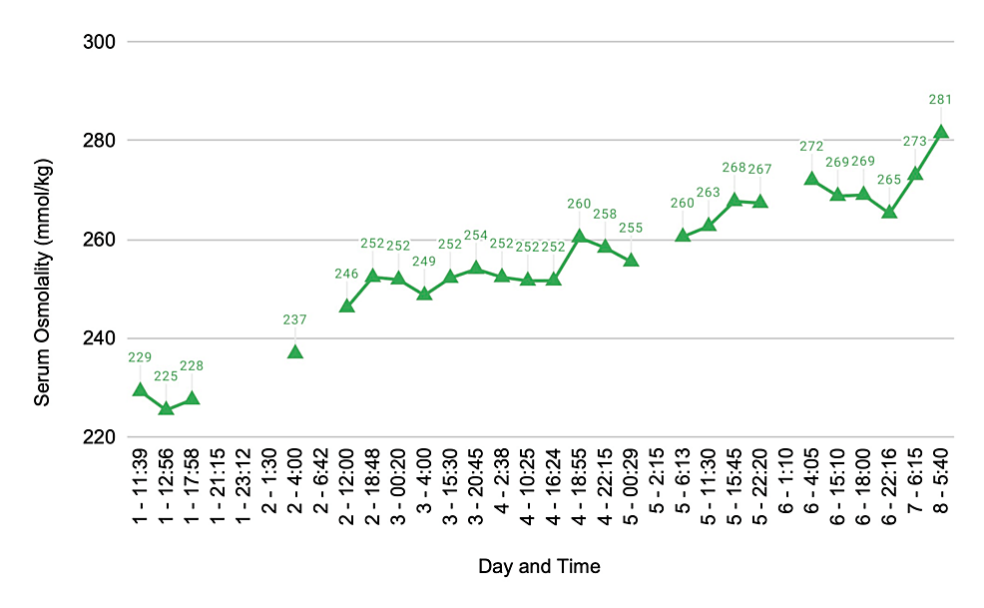
Serum osmolality (mmol/kg) from day one of admission to day eight of discharge

Over the next week, the patient's serum sodium gradually increased until he was discharged with stable serum sodium of 130 mEq/L (Figure 1) and a serum osmolality of 281 mmol/kg (Figure 2).

## Discussion

It has been widely reported that a side effect of substances of abuse, such as amphetamines, is hyponatremia. However, the toxicity of Kratom remains poorly understood and underreported. This patient's case indicates an association between Kratom and hyponatremia. Kratom is an herbal extract from a tree in Southeast Asia and has physiological effects similar to opioids, including pain relief, euphoria, and stimulant properties. However, there is still uncertainty regarding the safety of ingesting Kratom products. Kratom consists of a cocktail of psychoactive alkaloids occurring naturally in the plant, the most abundant being mitragynine. The primary metabolism of Kratom alkaloids involves the hepatic enzyme CYP3A4. Kratom also demonstrates linear pharmacokinetics and a biphasic elimination pattern, which explains how the excretion of the drug is determined by the amount released into the blood from the tissues. In addition, the half-life of mitragynine has been reported to be three hours, but some studies suggest it can be much longer [6].

Current research indicates Kratom acts on opioid receptors and may explain its association with developing hyponatremia. There are three subtypes of opioid receptors; Mu, Kappa, and Delta. Kratom was found to have a higher affinity towards the Kappa opioid receptors rather than the Mu and Delta receptors [7]. Chemically it acts as an agonist at the Mu receptors and an antagonist at the Kappa receptors. These effects lead to the inhibition of gamma-aminobutyric acid (GABA), an inhibitory neurotransmitter. By blocking the inhibitory actions of GABA, there is an increase in the secretion of antidiuretic hormone (ADH), which leads to hyponatremia [8].

When the patient's serum sodium was overcorrected by 4 mEq/L after the first day, the 3% sodium chloride was discontinued to decrease the risk of developing osmotic demyelination syndrome (ODS). This complication of overcorrecting hyponatremia leads to brain cell dysfunction from the destruction of nerve cells. In hyponatremia, there is a loss of osmotically active organic osmolytes from astrocytes that protect brain cells from swelling. When hyponatremia is corrected, the brain volume begins to shrink, and the organic osmolytes cannot be replaced as quickly. Therefore, a fall in brain volume results in demyelination due to direct injury to astrocytes and oligodendrocytes. In addition, cell water is lost after rapid correction, and potassium and sodium are shifted back into the cells. This effect results in DNA fragmentation, protein aggregation, and an increase in markers of cell death. Astrocytes and oligodendrocytes begin to die and release inflammatory cytokines [9-12].

This case report illustrates the effects of Kratom on the development of hyponatremia. It is critical to understand the severity of irreversible brain dysfunctions that can occur from hyponatremia, and especially important to act quickly in extreme cases such as this patient's presentation of serum sodium of 103 mEq/L.

## Conclusions

After excluding all other causes, the patient was determined to have hyponatremia-induced AMS secondary to the ingestion of Kratom. Without aggressive management, severe irreversible neurological complications would have occurred. This case presentation calls attention to Kratom’s poorly understood toxic profile and points out its need to be considered when other causes of hyponatremia have been ruled out.
